# Cocreation of Digital Outcome Measures for Dravet Syndrome: Multistage Co-Design Feasibility Study

**DOI:** 10.2196/92512

**Published:** 2026-09-25

**Authors:** Navdeep Sahota, Simona Giorgi, Ana Cantó Martínez, Naiara Sánchez Marco, Elisa Ferrer Mallol, Elin Haf Davies, José Ángel Aibar

**Affiliations:** 1 Aparito Ltd, Wrexham, United Kingdom; 2 Fundación Síndrome de Dravet, C/ Toledo, 46, 1º, Madrid, Madrid, 28005, Spain, 34 622883558

**Keywords:** Dravet syndrome, clinical outcome assessment, cocreation, public and patient involvement, encephalopathy, participatory research, caregivers

## Abstract

**Background:**

Dravet syndrome is a complex developmental and epileptic encephalopathy characterized by treatment-resistant seizures and multiple comorbidities that significantly affect quality of life. Traditional clinic-based assessments often fail to capture real-world functional abilities and behavioral changes.

**Objective:**

This study aimed to (1) identify caregiver-prioritized meaningful aspects of health, (2) co-design digital assessment modalities for home use, and (3) generate preliminary usability and implementation insights to inform future clinical research.

**Methods:**

A multistage patient and public involvement activity was conducted between November 2023 and October 2025 in Spain, in collaboration with a patient advocacy organization and a digital health company. Participants were recruited through the Fundación Síndrome de Dravet using a convenience opt-in sampling approach. The process included a caregiver survey to identify meaningful aspects of health, a design workshop to refine priorities and technology preferences, and a 2-week usability testing of a prototype app, followed by a feedback workshop. Data were analyzed descriptively to inform iterative cocreation; no hypothesis testing was performed.

**Results:**

Fifty caregivers completed the survey. Neuropsychiatric symptoms (17/48, 35%), independence (16/48, 33%), and social or leisure activities (15/48, 31%) were the most commonly reported affected domains. Eight caregivers participated in the design workshop, emphasizing flexibility, age-appropriate tasks, and reduced reporting burden. Usability testing was conducted with 5 caregivers over 2 weeks, with 4 of 5 caregivers providing feedback. Participants reported a generally positive reception of the digital tools, particularly customizable task selection and open-text fields, while identifying challenges related to video-recording logistics and questionnaire repetition. Feedback underscored the need for simplified workflows and individualized approaches to maintain engagement.

**Conclusions:**

Cocreation with caregivers is feasible and appears essential for developing meaningful digital outcome measures for Dravet syndrome. Video-based tasks and remote reporting tools show promise for capturing motor, cognitive, and behavioral domains beyond seizure frequency. Future work should focus on iterative refinement and formal validation of these measures as end points in clinical trials, ensuring that they reflect outcomes that matter most to patients and families.

## Introduction

Dravet syndrome (DS) is a complex developmental and epileptic encephalopathy characterized not only by treatment-resistant seizures but also by pervasive neurodevelopmental, motor, and behavioral comorbidities that drive the day-to-day burden for families [1-6]. Clinic-based assessments, typically episodic and symptom-siloed, often fail to capture meaningful changes in cognition, behavior, autonomy, and participation in real-world contexts. As a result, outcomes that matter most to families, such as independence, communication, and emotional well-being, remain undermeasured and underrepresented in decision-making [7-12]. Despite growing interest in digital health technologies to capture performance in naturalistic settings, there remains a paucity of patient-relevant, DS-specific outcomes that are (1) feasible at home, (2) sensitive to change beyond seizures, and (3) acceptable to caregivers across ages and abilities [13-15].

Digital outcome measures have already been used to evaluate motor function outside a clinical setting [13-16], have been proved to be feasible, and are used across a broad range of diseases, including in some clinical trials [17,18]. However, few outcome measures have been robustly validated and formally qualified as primary or secondary end points in clinical trials [17].

This paper reports a multistage patient and public involvement (PPI) cocreation activity designed to inform the early development and future validation of digital outcome measures rather than to test predefined hypotheses. Food and Drug Administration and European Medicines Agency guidance [19,20] on the use of digital health technologies [21] in clinical investigations informed key design decisions, including the prioritization of patient-relevant outcomes, the use of home-based assessment modalities, and attention to participant burden. Patient-focused development frameworks supported the iterative refinement of digital features to align with caregiver priorities and early considerations of future validation and trial readiness.

The determination of what to measure and how to measure it must begin with patients and caregivers so that concepts of interest (COI), modalities, and usage burden align with real-world needs. A PPI approach is, therefore, essential to co-define meaningful aspects of health (MAH) and co-design usable tools that families are willing to adopt [22-24]. In this study, we present a PPI activity involving collaboration between Fundación Síndrome de Dravet (FSD) in Spain and Aparito Ltd (UK). The aim of this PPI activity was to collaboratively cocreate digital outcome measures for DS with caregivers while generating early insights into feasibility, acceptability, and practical implementation. Specifically, we sought to (1) identify caregiver-prioritized MAH, (2) co-design digital assessment modalities suitable for home use, and (3) generate early usability and implementation insights to inform future clinical research and end point development.

## Methods

### Study Design

We conducted a multistage PPI activity in collaboration with a patient advocacy organization (FSD, Spain) and a digital health company (Aparito Ltd, UK). The activity was carried out remotely using online surveys, virtual workshops, and home-based usability testing of a prototype app.

#### Design and Framework

The study was guided by Aparito Ltd’s Patient Group Accelerator Program framework, which was developed to address unmet needs in rare disease patient groups. The framework incorporates principles of patient engagement, in addition to regulatory guidance proposed by the Food and Drug Administration and the European Medicines Agency, as well as recommendations from the Clinical Trials Transformation Initiative (CTTI) and the Digital Medicine Society on the development of patient-centric digital measures. The cocreation process followed a multistage approach, including a survey, a design workshop, usability testing, and a feedback workshop. A conceptual framework for quality of life (QoL) in DS was subsequently developed as a synthesis of findings from the literature review, survey, and design workshop, capturing key domains prioritized by caregivers and informing the interpretation of the results.

Using surveys and workshops with patient group representatives and caregivers, we aimed to ascertain the MAH, COI, and subsequent outcomes to be measured in a multistage cocreation approach.

Participant numbers at each stage were determined pragmatically, reflecting the exploratory PPI nature of the activity and the sequential, opt-in recruitment across stages. All survey respondents were invited to participate in subsequent stages, with participation declining at each step based on availability, interest, and scheduling constraints rather than predefined quotas.

Each phase of the activity was designed to build sequentially on the outputs of the previous one. Survey findings were summarized and presented to workshop participants at the start of the design workshop, providing a quantitative basis for discussion. Caregivers were asked to reflect on whether the survey results resonated with their own experience, to add nuance to or challenge the priorities identified, and to discuss technology preferences and measurement modalities in greater depth. The workshop thus served to qualitatively validate and refine the survey findings rather than replicate them.

The outputs of the design workshop, including agreed-upon priority domains, preferred activities of daily living (ADL) tasks, and caregiver input on app features such as the seizure diary structure, task flexibility, and questionnaire format, were translated directly into the configuration of the Atom5 prototype app used in usability testing. Specifically, the task list presented to usability testing participants reflected the ADL tasks discussed and endorsed during the workshop, the seizure diary form incorporated the fields and free-text options requested by workshop attendees, and the QoL questionnaire’s frequency and format were adjusted in line with caregiver feedback on reporting burden. Usability testing then provided a first empirical test of whether these co-designed features were feasible and acceptable in a real home-use context over 2 weeks.

All the components were not administered as research outcome measures, and their outputs were not subjected to quantitative analysis. Rather, they served as experiential probes to generate caregiver feedback on the feasibility, usability, and acceptability of each feature in a real home-use context. Findings are therefore reported qualitatively as caregiver perceptions of each component rather than as performance or adherence metrics.

#### Study Timeline

The activity was conducted between November 2023 and April 2025. An initial agreement between Aparito Ltd and FSD was established in November 2023 to initiate the cocreation process. The caregiver survey was launched in January 2024 and remained open for 2 weeks (until January 31, 2024). A design workshop was conducted in May 2024 to refine priorities and explore technology preferences.

Between September 2024 and December 2024, a prototype app was developed, including the configuration of questionnaires, seizure diary features, and video-based tasks informed by the earlier phases. Usability testing was conducted between January 2025 and March 2025, with a 2-week testing period for each participant. A final feedback workshop was held in April 2025, after which the project was closed. The sequence and timing of the study activities are illustrated in Figure S1 of Multimedia Appendix 1.

### Survey

A caregiver survey was developed to explore MAH, COI, and technology acceptance [25]. Items were adapted from the CTTI Question Bank for Identifying Meaningful Outcome Measures to ensure relevance to DS and feasibility for remote completion [26]. Questions were adapted for language simplification, removal of clinically interpretive items, and the addition of DS-specific examples. Final item selection was agreed upon jointly by patient representatives from FSD and researchers at Aparito Ltd.

Inclusion criteria for the study were (1) being of legal age (≥18 y), (2) residing in Spain, and (3) being a caregiver of a patient with a clinical diagnosis of DS. Informed consent was obtained from all participants. Potential participants were identified and contacted through the established caregiver network of FSD, including its mailing list, online support groups, and social media channels. No purposive or stratified sampling was applied; the survey was distributed broadly to all caregivers registered with or known to FSD. No minimum number of responses was predefined. Eligibility was based on self-identification as a caregiver of an individual with DS and on recruitment through FSD. The majority of participants were already engaged with FSD through support programs, newsletters, or prior interactions, which provides a high level of confidence in caregiver status within the context of service development and cocreation activities. Survey items were not mandatory to reduce respondent burden and allow caregivers to skip questions they considered not applicable. The survey is provided in Multimedia Appendix 1. It consisted of 23 open-ended and structured items organized into 5 domains: MAH, COI, outcomes to be measured, demonstration of therapeutic benefit, and technology acceptance. The survey was administered online through a secure web-based platform and distributed by FSD via its communication channels (eg, newsletters and direct outreach). Responses were collected anonymously, and participants could skip questions to minimize burden.

### Design Workshop

A design workshop was conducted to gather qualitative insights and validate survey findings regarding technology modalities. Participants for the design workshop were recruited from among the survey respondents who had expressed willingness to participate in further activities. FSD staff contacted eligible individuals directly via email. Selection was guided by the age of the patient cared for and the area of residence to ensure a balanced distribution across age ranges and geographic locations. Eight caregivers participated. The discussion was facilitated by members of the research team with experience in patient engagement. The session was conducted using semistructured, open-ended prompts to elicit caregiver perspectives on priorities, usability, and technology preferences and lasted for 1.5 hours. The meeting was recorded and automatically transcribed using the built-in transcription feature of Microsoft Teams. Transcripts were subsequently reviewed and manually corrected by a member of the research team to ensure accuracy prior to thematic synthesis. Responses were synthesized thematically through iterative team discussion involving all members of the research team present at the session. Following transcription, each team member independently identified recurring concepts from the transcript before a joint discussion was held to compare observations, resolve discrepancies, and reach consensus on the final themes. Reflexivity was considered informally throughout the process, with team members acknowledging their dual roles as both researchers and members of the patient advocacy organization and actively discussing how this positionality might influence theme identification. Themes were required to reflect concepts raised by multiple participants independently rather than isolated comments to minimize the risk of researcher-driven interpretation. No formal qualitative coding software was used, consistent with the exploratory and hypothesis-generating nature of this PPI activity. Following the design workshop, the codeveloped inputs were translated into a prototype app configured within the Atom5 platform (Aparito Ltd). At the end of the 2-week period, participants provided feedback through a structured user experience survey administered within the app.

### Conceptual Framework

The development of the framework was led by members of the research team at Aparito Ltd, including researchers with experience in patient engagement and digital health. Survey results were reviewed descriptively to identify caregiver-prioritized domains, while qualitative insights from the design workshop were examined to capture recurring themes related to daily functioning and QoL. These inputs were iteratively discussed within the study team to group related concepts into broader domains and structure the relationships between them. The resulting framework reflects a pragmatic synthesis of quantitative and qualitative findings, rather than a formal qualitative analysis, and was used to support the interpretation of the cocreation outputs.

### Usability Testing

The purpose of the usability testing was to assess the feasibility, acceptability, and perceived usability of the co-designed prototype app in a real home-use context and to identify practical barriers and facilitators to engagement with each app component prior to any future formal validation study. A structured 2-week usability testing period was conducted to explore practical feasibility, caregiver experience, and usability of the codeveloped prototype app (Atom5). Five caregivers identified by FSD participated. Upon providing informed consent, participants received a QR code to download the app and were guided through onboarding via an in-app information module. Daily push notifications were sent to prompt the completion of tasks and questionnaires.

Caregivers were asked to complete 3 predefined components during the testing period: (1) a daily seizure diary, structured as a stepwise form with a yes or no question on seizure occurrence and optional fields for seizure type, duration, mood, location, triggers, event description, and postevent status; (2) up to 3 video-based ADL tasks selected by each caregiver from a codeveloped list (Table S1 in Multimedia Appendix 1) according to the patient’s abilities and preferences, with standardized filming instructions provided in Spanish; and (3) bespoke QoL questionnaires for patients and caregivers, developed by drawing on validated instruments, including the EQ-5D-5L (EuroQol 5-dimension 5-level) and the DANCE (Dravet Syndrome-Associated Neuropsychiatric Comorbidities Evaluation; Checklist 1) checklist [27]. Questionnaire items were not directly adapted from these instruments but were newly developed by the research team, drawing on the conceptual domains identified in both the literature review and the caregiver survey. The established instruments served as a reference framework to ensure content validity and alignment with previously validated constructs rather than as a source of verbatim or modified items. Video recordings could be captured directly within the app or recorded externally and uploaded to the platform. All videos were stored only for the duration of the testing period on a secure, encrypted platform, with access restricted to a single designated researcher. These instruments were developed specifically for this activity and were not formally pretested or psychometrically validated prior to use, which is consistent with the exploratory PPI nature of the activity.

At the end of the 2-week period, participants completed a structured user experience survey administered through the app that assessed ease of onboarding, app usability, clarity of instructions, comfort with video recording, and perceived usefulness of features such as voice activation and push notifications.

Given the exploratory and hypothesis-generating nature of the activity, the primary goal of usability testing was to assess caregiver experience, perceived usability, and willingness to engage with the platform rather than quantify adherence or generate performance benchmarks. Subjective caregiver feedback was therefore prioritized as the main data source, consistent with the PPI framing of the activity. Future studies with a formal feasibility or validation design should incorporate objective engagement metrics as predefined end points.

Caregivers who had participated in the survey were invited to take part in usability testing. Given the exploratory nature of the activity, no formal selection criteria were applied beyond prior participation and willingness to engage with a smartphone app for a 2-week period. FSD staff coordinated the recruitment through direct contact.

### Feedback Workshop

A final feedback workshop was conducted remotely via video conference to gather in-depth reflections on the usability testing experience. The session was facilitated by members of the FSD and Aparito research teams and followed a semistructured discussion guide based on the findings from the end-of-period user experience survey (Multimedia Appendix 1), covering topics such as task feasibility, seizure diary usability, questionnaire burden, video-recording challenges, and the overall app experience. The session was not formally recorded; notes were taken by a member of the research team during the session. FSD invited all 5 caregivers who had completed usability testing to participate in the feedback workshop via direct email. No additional selection criteria were applied. The meeting lasted 1 hour.

Each stage (Table 1) built on the previous one, allowing iterative refinement based on caregiver feedback. Objective usage metrics, such as task completion rates, submission frequency, or time on task, were not systematically collected during this phase.

**Table 1.** Stages of involvement throughout the patient and public involvement activity.

Stage	Activity	Participants	Role and level of involvement	Impact on design
Survey	Identify MAH^a^	50 caregivers	Consultation	Prioritized neuropsychiatric symptoms and independence
Design workshop	Validate priorities and discuss tech	8 caregivers	Collaboration	Selected video tasks and simplified diary
Usability testing	Test prototype app	5 caregivers	Partnership	Requested open-text fields and flexible task list
Feedback workshop	Reflect on experience	2 caregivers	Partnership	Recommended reduced questionnaire frequency

a MAH: meaningful aspects of health.

### Ethical Considerations

This activity was prospectively designed and conducted as a PPI activity rather than as biomedical research, and it does not constitute biomedical research under Spanish Law 14/2007 on Biomedical Research; therefore, formal research ethics committee approval was not required. No clinical interventions were performed, and no identifiable personal data were collected. All participants provided informed consent prior to participation and were informed about the purpose of the activity, the voluntary nature of their involvement, the types of data collected (including optional video recordings), data storage duration, restricted access procedures, and the intention to disseminate aggregated findings in a peer-reviewed publication. Participants did not receive financial compensation or material incentives. Data handling complied with the European Union General Data Protection Regulation (2016/679) [28] and the Spanish Organic Law 3/2018 on Data Protection and Digital Rights [29]. Throughout the activity, ethical principles of transparency, informed consent, proportionality, and data minimization were applied in accordance with the recommendations of the Spanish Code of Good Practices in Health Research (Instituto de Salud Carlos III) [30].

## Results

### Survey Findings

A caregiver-targeted survey was conducted over a 2-week period in January 2024. The survey included 5 thematic sections: MAH, COI, outcomes to be measured, demonstration of therapeutic benefit, and technology acceptance, incorporating questions adapted from the CTTI Question Bank for Identifying Meaningful Outcome Measures. A total of 50 caregivers responded; however, not all respondents answered every question, as completion of individual items was optional. A summary of the results is provided in Table 2.

**Table 2.** Summary of caregiver survey findings (n=50; not all respondents answered every item).

Domain and key finding	Values, n/N (%)
Most impacted areas (patient)	
Neuropsychiatric symptoms	17/48 (35)
Independence	16/48 (33)
Social and leisure activities	15/48 (31)
Most impacted areas (caregiver)	
Loss of independence	19/47 (40)
Reduced social or leisure activities	18/47 (38)
Totally affected (patient)	
Independence	25/48 (52)
Totally affected (caregiver)	
Independence	21/48 (44)
Barriers to daily activities	
Neuropsychiatric symptoms	22/45 (49)
Motor or physical limitations	19/45 (42)
Desired improvements	
Seizure reduction	16/42 (38)
Communication	12/42 (29)
Attention and cognition	11/42 (26)
Priority for judging treatment benefit	
Seizure reduction	35/44 (80)
Neuropsychiatric improvement	16/44 (36)
Technology acceptance	
Digital tools useful for clinical measurement	25/40 (63)
Willingness to use smartphone app	34/42 (81)
Good tolerance of wearables	31/45 (69)

Findings from the survey addressed aim 1 by identifying caregiver-prioritized MAH. Fifty caregivers completed the survey; however, not all respondents answered every item, as completion was optional. Key findings are summarized in Table 2.

Neuropsychiatric symptoms, independence, and social participation emerged as the domains most impacted by DS for both patients and caregivers. Independence was reported as totally affected by more than half of caregiver respondents for their care recipients and by nearly half for themselves. The primary barriers to daily activities were neuropsychiatric symptoms and motor or physical limitations.

Regarding treatment priorities, seizure reduction was overwhelmingly the most valued outcome, followed by neuropsychiatric improvement. When asked what could be measured at home, respondents most frequently selected seizures, the emotional impact on the family, and performance in daily tasks. Technology acceptance was high: the majority of respondents viewed digital tools as useful for clinical measurement, and their willingness to use a smartphone app for symptom reporting was particularly strong. Wearable devices were generally accepted, although concerns about tolerability were noted by a minority of respondents.

### Design Workshop

**Figure 1.**
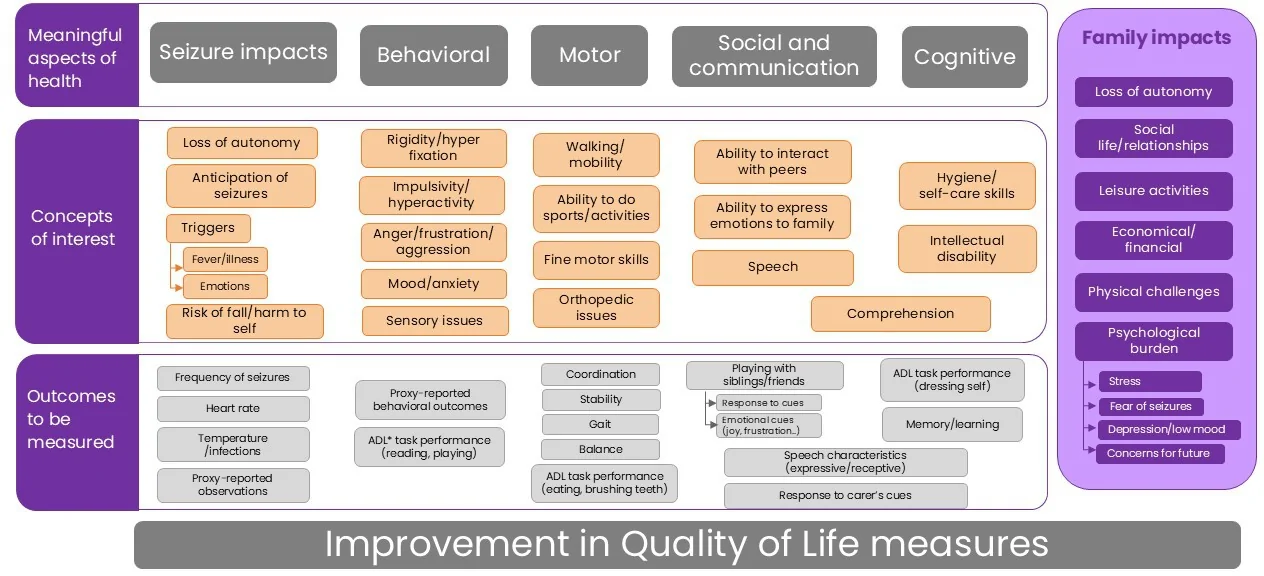
Conceptual framework of quality of life in Dravet syndrome (DS) obtained from the literature review, incorporating meaningful aspects of health identified for patients with DS and their families, and responses to the questionnaire and design workshop. ADL: activities of daily living.

Following the survey, 8 caregivers participated in a workshop to gather qualitative insights, validate the survey findings, and discuss technology preferences. Findings from the design workshop addressed aim 2 by informing the co-design of digital assessment modalities. These findings also contributed to the development of a conceptual framework summarizing key domains of QoL in DS (Figure 1) as a synthesis of the survey results and themes identified during the design workshop. Caregivers highlighted the importance of cognitive issues, although these were noted to fluctuate depending on various factors, such as the patient’s age, mood, and time of day. It was also mentioned that cognitive and social afflictions were often accepted as something that cannot be changed. Moreover, caregivers expressed concerns about the onset of puberty, which could cause potential exacerbation of the disease and changes in the child’s behavior or cognition due to hormonal changes. A subset of participants expressed a more immediate focus on motor development and speech, given the young age of their children.

Despite these differences, the group converged on a shared set of MAH that was broadly consistent with the domains represented in the conceptual framework of QoL in DS (Figure 1). This convergence was assessed informally: themes were considered to reflect group agreement when raised independently by multiple participants without substantive objection from others, consistent with the exploratory and nonformal nature of this PPI activity.

When asked about their willingness to use digital tools, participants saw value in being able to monitor health variables, especially in the context of DS, which requires a high level of monitoring by caregivers. However, concerns were raised about the practicality of wearing devices, the accuracy of measurements, especially in the case of movement, and the cost-effectiveness of certain products. The idea of using these devices at specific time points, such as during a clinical trial rather than continuous monitoring, was also suggested. Among the tasks and activities that could be valuable to collect in a clinical trial were monitoring sleep patterns during the day and at night, tracking autonomy and movement around the house, and assessing fine motor skills. The ability to walk, and how this varied throughout the day and with mood, was also identified as a potentially valuable measure.

Overall, while participants see the potential benefits of digital tools, they also highlight the need for reliable, accurate, and user-friendly devices and the importance of measuring both physical and cognitive aspects in clinical trials.

During the workshop, attendees provided input on the design features:

- Seizure diary was designed as an in-app feature with a simplified input form that captures whether a seizure occurred (if not, the user can submit and return to the homepage), seizure type (according to the most recently published International League Against Epilepsy seizure classification [25]), duration, mood, location, triggers, event description (in lay terms), and postevent status.
- Task selection and video tasks were designed to capture neuropsychiatric aspects affecting ADLs, such as eating, playing, dressing, and communicating, based on the activities discussed by caregivers during the workshop. The cocreated tasks reflect relevant ADLs for patients with DS across age groups (Table S1 in Multimedia Appendix 1). Video-based assessments may provide a more objective alternative for individuals unable to complete patient-reported outcomes, such as young children or those with severe disabilities [16,31].

The prototype comprised 4 main modules informed directly by workshop outputs: (1) a screening questionnaire collecting basic demographic information (caregiver gender and age, relationship to the individual with DS, and the patient’s age), used to verify eligibility and contextualize responses; (2) a bespoke caregiver QoL questionnaire, administered on a recurring basis, including items rated on 0 to 10 numeric scales (eg, agreement with statements about the patient’s condition; perceived change relative to baseline ranging from “much worse” to “much better”) and multiple-choice items (eg, seizure frequency over the past 4 weeks: stable, improvement, worsening, or unknown). Items were presented in Spanish; (3) a daily seizure diary, structured as a stepwise form starting with a yes or no question on seizure occurrence, followed by optional fields for seizure type, duration, mood, location, triggers, event description, and postevent status; (4) a video task module, presenting a codeveloped list of ADL tasks from which caregivers could select up to 3 according to the patient’s abilities and preferences, with standardized filming instructions provided in Spanish.

The app was configured in Spanish throughout and included an onboarding module, daily push notifications, and a final user-experience survey administered at the end of the 2-week period.

### Usability Testing

Findings from the usability testing and the feedback workshop addressed aim 3 by providing preliminary insights into usability and implementation. Usability testing was conducted with 5 caregivers identified by FSD (3 mothers and 2 fathers) over a 2-week user-testing period using a prototype app configured within Atom5.

Four participants completed the survey. The platform received positive feedback regarding the onboarding process, app usability, and the feasibility of using the app at home to collect data. There was some negative feedback regarding task recordings being challenging to get a clear video and requiring 2 people, in addition to not receiving or finding the push notifications to be useful (some testers may not have allowed them when onboarding onto the app accidentally). The general feedback was positive, with 1 participant mentioning that the questionnaire felt a bit repetitive. These points were followed up during the feedback workshop.

### Feedback Workshop

The caregivers who tested the app were invited by FSD to participate in a workshop to discuss their experience with the app. Due to scheduling difficulties, only 2 caregivers attended; both were parents of the same adult individual with DS. Feedback from the 2 caregivers highlighted the need for flexibility and adaptability in task design and data entry. Some tasks were perceived as too simple or too brief, whereas others varied in difficulty depending on the individual’s cognitive and motor abilities, underscoring the importance of allowing task selection and adaptation. Caregivers valued the ability to modify or switch tasks when activities were neither engaging nor feasible, supporting the need for a broad and customizable task list.

Participants also emphasized flexibility in seizure reporting and requested open-text fields and skip options when predefined categories did not adequately capture seizure characteristics, triggers, or mood changes. Repetitive data entry for frequent or similar seizures was described as burdensome, with suggestions to reduce reporting fatigue through more efficient input options.

Concerns were also raised regarding the questionnaire frequency, with caregivers indicating that overly frequent administration could be tiring and demotivating, particularly given the slow pace of change in QoL. Finally, the recording of video tasks was reported as challenging in some situations due to attention limitations, the need for caregiver assistance, and technical constraints, highlighting the importance of flexible recording requirements and clear guidance [32].

Objective usage metrics, such as task completion rates or submission frequencies, were not systematically collected during this exploratory usability phase.

### Impact of PPI on Design

Caregiver input shaped multiple design decisions, including (1) a simplified input form and the addition of open-text fields for atypical triggers in the seizure diary; (2) an expanded task list for video recording to accommodate varying abilities, with the option to substitute tasks; (3) reduced frequency and added customizable intervals to minimize questionnaire burden; and (4) adjusted notification settings based on caregiver feedback about usability. These changes illustrate how PPI moved beyond consultation to active cocreation, ensuring that the prototype reflected real-world needs.

These recommendations are summarized in Table 3. Changes implemented in the initial prototype reflect co-design decisions made during the workshop phase, whereas recommendations arising from usability testing and the feedback workshop are intended to inform the next development iteration.

**Table 3.** Mapping of caregiver feedback to design recommendations across patient and public involvement stages.

Stage	Caregiver feedback	Design recommendation	Status
Design workshop	Need for flexible task selection according to patient age and ability	Codeveloped customizable ADL^a^ task list; caregivers select up to 3 tasks	Implemented in prototype
Design workshop	Simplified seizure reporting to reduce burden	Stepwise form with yes/no entry point and optional fields	Implemented in prototype
Usability testing/feedback workshop	Questionnaire frequency perceived as repetitive and tiring	Reduce questionnaire administration frequency; consider quarterly completion	Recommended for next iteration
Usability testing/feedback workshop	Push notifications not received or enabled during onboarding	Review onboarding flow to ensure notification permissions are explicitly requested	Recommended for next iteration
Usability testing/feedback workshop	Video recording challenging without a second person; need for flexibility	Expand task list; allow task substitution; clarify filming instructions	Recommended for next iteration
Usability testing/feedback workshop	Open-text fields needed for atypical seizure triggers	Add free-text option to seizure diary trigger and description fields	Recommended for next iteration

a ADL: activities of daily living.

## Discussion

### Principal Findings

This multistage PPI activity demonstrated that caregivers of individuals with DS can actively contribute to the identification, prioritization, and early design of digital outcome measures intended for home-based use. Across the survey, workshop, and usability testing stages, caregivers consistently emphasized neuropsychiatric symptoms, independence, and daily functioning as meaningful domains, and highlighted the importance of flexibility, reduced reporting burden, and adaptability to the wide variability in abilities, which aligns with recent findings [33,34].

This activity was designed as a hypothesis-generating PPI activity rather than as hypothesis-driven biomedical research, which limits inferential interpretation but is appropriate for early cocreation and feasibility assessment. The Aparito Patient Group Accelerator Program provided the framework for involving patients and caregivers starting from the initial stage of codevelopment for new digital outcome measures, ranging from the identification of the MAH to content design and final user testing [21].

This cocreation approach is particularly relevant for complex conditions such as DS, where patient experiences are highly variable and multifaceted [32]. Involving caregivers from the outset is essential to ensure that the tools developed are meaningful, usable, and aligned with real-world needs. The activity illustrated that the impact of DS on daily life varies considerably across patients and families, reflecting the heterogeneous and multifaceted nature of the condition, and confirming previous results [12,35].

Caregivers’ input influenced priorities (neuropsychiatric symptoms and independence), technology choices (video tasks and simplified diaries), and usability features (open-text fields and flexible task lists). Digital tools were perceived positively by participating caregivers, a finding consistent with previously published findings [36,37], although conclusions are limited by the small, self-selected sample [32,35]. The bespoke QoL questionnaires developed in this activity represent the first iteration of a cocreated instrument and were not designed for immediate use as validated end points. Future work should focus on formal psychometric validation, including assessment of content validity, test-retest reliability, and responsiveness to change, with a view to qualifying the instrument as a patient-reported outcome measure suitable for use in clinical trials.

Feedback from the seizure diary component emphasized the importance of flexibility in reporting. Although seizure diaries have been widely used in DS to monitor seizure frequency and duration [38,39], caregivers in our pilot highlighted the limitations of rigid categorization and the need for open-text fields to better capture individualized triggers and seizure types. Regarding QoL assessments, caregivers suggested that quarterly completion would be optimal to avoid fatigue and disengagement.

The feedback gathered underscores the importance of iterative design, caregiver involvement from concept generation to design and testing, and contextual sensitivity in digital health innovation. These findings inform both the future development of digital platforms and clinical trial design and contribute to broader efforts to create meaningful, patient-centered digital outcome measures for rare and complex conditions.

The feasibility of conducting meaningful PPI with caregivers of individuals with DS warrants substantive reflection, as the process itself generated important learnings beyond the design outputs. Recruitment through FSD’s established caregiver network facilitated initial engagement and provided a high level of confidence in participant eligibility but also introduced a degree of selection bias toward more engaged and digitally receptive families. Retention across stages declined substantially, from 50 survey respondents to 8 workshop participants, to 5 usability testers, and finally to 2 feedback workshop attendees. This attrition pattern is consistent with what has been reported in PPI activities with caregivers of individuals with complex rare conditions, where competing caregiving demands, fatigue, and scheduling constraints are pervasive barriers to sustained participation [40-42]. Caregivers of individuals with DS, in particular, carry substantial psychosocial and practical burdens that may limit their availability for multistage research engagement [43].

Several elements appeared to support engagement in the earlier stages of the activity. The survey format was well suited to this population: it was brief, remote, optional in terms of item completion, and distributed through a trusted advocacy organization. The design workshop benefited from being conducted as a group session, which created a sense of shared experience among participants and facilitated richer discussion than individual interviews might have generated. Caregivers responded positively to seeing their survey results presented back to them at the start of the workshop, which reinforced the sense that their input was valued and acted upon.

However, sustaining engagement across the full multistage process proved challenging. The 2-week usability testing period required a substantial and sustained time commitment from caregivers who already carry high daily burdens. The low attendance at the feedback workshop, despite flexible scheduling and multiple reminders, suggests that even well-intentioned accommodations may be insufficient when competing with the daily realities of caring for an individual with DS. In retrospect, a shorter or asynchronous feedback format, such as a brief structured questionnaire or a recorded video response, might have achieved higher participation at this stage while preserving the depth of feedback needed.

These findings have direct implications for the design of future PPI activities and cocreation studies in this population. We would recommend (1) front-loading the most critical co-design decisions to the earliest, highest-participation stages; (2) offering asynchronous participation options at later stages to reduce scheduling barriers; (3) considering staged incentivization or recognition of caregiver contributions to support retention; and (4) building explicit dropout contingency plans into multistage PPI designs, particularly for final validation or feedback stages. More broadly, the attrition observed across stages should not be interpreted solely as a methodological limitation but also as a substantive finding about the feasibility of sustained PPI engagement in this population that may be transferable to other rare disease contexts with similar caregiver burden profiles.

### Limitations

This activity has several limitations. First, participant numbers decreased substantially across stages, with only 2 caregivers attending the final feedback workshop despite all 5 usability testing participants being invited and reminded on multiple occasions. Both attendees were parents of the same adult individual with DS, which limits the diversity of perspectives captured at this stage and introduces a risk of homogeneity bias in the final feedback. This attrition likely reflects the considerable competing demands faced by caregivers of individuals with complex conditions, for whom participation in multistage research activities can be difficult to sustain over time. Proactively addressing these barriers, for example through shorter or asynchronous feedback formats, more flexible scheduling, or dedicated caregiver support, will be important in future cocreation studies with this population. The limited and homogeneous participation in the feedback workshop should be considered when interpreting the design recommendations that emerged from this stage. Second, caregiver status was based on self-report and recruitment through FSD rather than independent clinical verification, which may introduce selection bias toward more engaged or digitally receptive caregivers. The sample, therefore, probably does not represent the full spectrum of DS caregivers, and findings on acceptability and usability may be more optimistic than would be observed in a broader or less self-selected population. Third, usability testing was short in duration and did not include objective usage metrics, such as task completion rates or adherence over time, as this was not the purpose of the testing. Finally, the activity was conducted in a single language and geographic context, which may limit cultural transferability.

Participant demographic and clinical characteristics were not systematically collected across all stages of the activity, in line with the data minimization principles applied throughout. As a result, it is not possible to characterize the sample in terms of caregiver relationship, patient age range, or digital familiarity, which limits readers’ ability to contextualize the findings and assess their broader applicability. Future cocreation studies with this population should consider collecting a minimal set of anonymized descriptors, such as caregiver relationship, broad patient age group, and self-reported digital experience, to strengthen interpretability without imposing undue burden on participants.

.

### Conclusions

This PPI activity suggests preliminary feasibility and the potential value of codeveloping digital outcome measures for DS through the active involvement of caregivers and patient advocates. By leveraging insights from the conceptual framework, we identified digital options to capture seizures, daily functional abilities, and QoL in the home environment. We addressed important gaps in current assessment tools, particularly the limited ability of clinic-based evaluations to reflect the real-world impact of neurodevelopmental and behavioral comorbidities. With this activity, video-based tasks have been identified as a promising approach to remotely observe motor, cognitive, and social behaviors that may not be easily measured using traditional scales or in the clinical environment.

This PPI activity contributes (1) a cocreated concept set of outcomes and home-based assessment modalities prioritized by caregivers; (2) usability and acceptability insights that translate caregiver preferences into concrete design requirements (eg, flexible task lists, open-text fields, and reduced reporting burden); and (3) a pathway for trial readiness, specifying how cocreated measures may be iteratively refined and prepared for future psychometric validation and regulatory alignment.

Future work should focus on iterative refinement of the tools and rigorous validation of digital end points in longitudinal studies and clinical trials. Ultimately, integrating cocreated digital measures into clinical research and care pathways may support a more comprehensive evaluation of emerging therapies by capturing changes that truly matter to individuals with DS and their families. 

## Supplementary material

10.2196/92512 Multimedia Appendix 1 Timeline, survey, and interview guide.

10.2196/92512 Checklist 1 DANCE checklist.

## Abbreviations

ADL: activities of daily living
COI: concepts of interest
CTTI: Clinical Trials Transformation Initiative
DANCE: Dravet Syndrome-Associated Neuropsychiatric Comorbidities Evaluation
DS: Dravet syndrome
EQ-5D-5L: EuroQol 5-dimension 5-level
FSD: Fundación Síndrome de Dravet
MAH: meaningful aspects of health
PPI: patient and public involvement
QoL: quality of life
