# Supplementary material for: Cocreation of Digital Outcome Measures for Dravet Syndrome: Multistage Co-Design Feasibility Study
Source: J Particip Med. 2026 Sep 25;18:e92512. doi: 10.2196/92512 (PMC13614624; doi:10.2196/92512)
Supplement: Supplementary file 1 [file jopm-v18-e92512-s001.docx]

# SUPPLEMENTARY FIGURES

Figure S1: Timeline of the multi-stage Patient and Public Involvement activity, illustrating the sequence and timing of study phases, including survey administration, design workshop, prototype development, usability testing, and feedback workshop.

# SURVEY

Thank you for taking the time to answer this survey. We are looking for parents and caregivers of children with Dravet syndrome to answer some questions on their views and experiences. The answers to these questions will help us to understand more what is important to families of children with Dravet syndrome.

We will not capture any personal information in this survey and all responses are anonymous. Please do not write any names or places in the answers. Please try to respond with as much detail as possible. You do not have to answer any questions you do not want to.

When you have completed the survey please press ‘Submit’.

**Meaningful Aspect of Health**

1.a. What part of **your child’s** life is most frustratingly impacted by their condition?

1.b. What part of **your and your family's** life is most frustratingly impacted by their condition?

2.a. How has **your child’s** life independence been affected by their condition?

2.b. How has **your** life independence been affected by their condition?

3.a. What about your child’s **physical** health do you wish you could improve?

3.b. What about your child’s **emotional aspects and/or behavioural symptoms** do you wish you could improve?

4. Considering what you just mentioned, explain your near-term goals for your child.

a .In the next **3 months** I’d like my child to (e.g. start or continue doing):

b. In the next **6 months** I’d like my child to be able to:

5. Explain your longer-term goals for your child.

In the next **12-18 months** I'd like my child to (e.g. start or continue doing):

**Concept of Interest**

1. What symptoms (or things about your child’s condition) impact their ability to do what you wish they could do? Provide an example.

2.a. On a given day, what are the symptoms that bother your child more than others? What makes these bothersome to your child?

2.b.On a given day, what are the symptoms that bother you more than others? What makes these bothersome to you?

3. What are your top 3 concerns about your child? (Please mention here specific symptoms, emotions or behaviours)

**Concern 1:**

**Concern 2:**

**Concern 3:**

**Outcome to be Measured**

1. Regarding the concerns you mentioned above, what could be measured to better understand if your child’s health is improving or not getting worse?

**Measure concern 1:**

**Measure concern 2:**

**Measure concern 3:**

2. Why would measuring this be a good way to track or understand your child’s condition?

**Measure concern 1:**

**Measure concern 2:**

**Measure concern 3:**

3.a. What has your doctor or health care provider measured to better understand your child’s condition?

3.b. Do these measures make sense to you? Please explain why/why not?

3.c. How useful do you think these measures are? Why do you feel that way?

4. What could be measured in your home environment that isn’t currently?

**Demonstrating Therapeutic Benefit**

1. How do you know when your child’s condition is improving?

2. Which changes would you want to see in your child’s symptoms before it really starts to make a positive difference in your child’s life?

3.a. How would this change	impact on your child’s life?

3.b. How would this change impact your life?

3.c. How would this change impact your family's life?

4. What would you need to see for you to feel that a pharmacological treatment is beneficial for your child?

**Technology acceptance**

1. Would the use of a digital technology to measure this change help? Why/why not?

2. Would you feel comfortable using an app on your smartphone or tablet to answer questionnaires about your child or report your child’s symptoms?

3. Would you feel comfortable using an app to send video recordings of your child doing certain tasks (e.g. walking, eating, talking periodically) to be reviewed and analysed by researchers and computer programmes?

4. Would your child be able to wear a device (e.g. smartwatch) that records physiological parameters passively in the background through the day?

5. Is there a moment of the day/night where you would prefer to perform measurements of your child’s activity? (e.g. recording video while eating, sleep monitoring etc…)

# INTERVIEW GUIDE

**1. Introduction and aims** *(~5 min)*

- Presentation of the PGAP framework and the purpose of the session: to identify meaningful aspects of health (MAH), concepts of interest (COI), and potential outcomes to measure in Dravet syndrome.

**2. Meaningful aspects of health** *(~15 min)*

- Do these results [survey findings] reflect your main concerns as a caregiver?
- Are there concerns you feel are missing or underrepresented?
- Do you think concerns differ depending on the age of the patient or other factors such as puberty or gender?

**3. Concepts of interest and outcomes to measure** *(~15 min)*

- What symptoms or aspects of the condition most impact daily life for your child and your family?
- What would you most like to be able to measure or monitor at home?
- What activities or tasks do you think could be captured digitally (e.g., sleep, motor function, autonomy, fine motor skills)?

**4. Overview of the Atom5™ platform and digital tools** *(~10 min)*

- Do these platform capabilities resonate with the aspects you would like to investigate?
- What are your thoughts on wearable devices? Would continuous monitoring be feasible or preferable at specific timepoints (e.g., during a clinical trial)?

**5. Activities of Daily Living (ADLs) and video task feasibility** *(~20 min)*

- Which daily activities do you think could be filmed and submitted as part of a study?
- How feasible would it be to follow standardized filming instructions at home?
- How often would you be willing to submit videos or complete questionnaires?
- Are there activities that would be easy or difficult to capture? (e.g., eating, walking, turning pages of a book, brushing teeth)
- Would flexibility in task selection and timing be important to you?

**6. Technology acceptance and data privacy** *(~10 min)*

- Would you be comfortable completing questionnaires through a smartphone app?
- Would you be comfortable recording and sharing videos for research purposes, given appropriate data security measures?
- What concerns, if any, do you have about data privacy?

**7. Open questions and closing** *(~5 min)*

- Is there anything else you would like to add or any aspect we have not covered?
