# Supplementary material for: Cocreation of Digital Outcome Measures for Dravet Syndrome: Multistage Co-Design Feasibility Study
Source: J Particip Med. 2026 Sep 25;18:e92512. doi: 10.2196/92512 (PMC13614624; doi:10.2196/92512)
Supplement: Supplementary file 2 [file jopm-v18-e92512-s002.docx]

# DANCE CHECKLIST

### COGNITION & BEHAVIOUR

**What is the level of cognition of the patient?**

**Language**

☐ Non-verbal (gestures, posture, eye-contact, facial expression)

☐ Few single words (less than 20 words)

☐ Simple language (able to produce short sentences, restricted number of words)

☐ Normal/Fluent (able to produce complex sentences, full aquisition of the vocabular)

Has the patient ever had a formal evaluation by a professional?

YES ☐	NO ☐	NOT SURE ☐

Has the patient had professional support for it in the last six months?	YES ☐	NO ☐	NOT SURE ☐

**Intelligence Quotient (IQ)* score (patient above 6 years of age):**
**IQ is a total score derived from several standardized tests designed to assess human intelligence. It indicates how far above or below an individual stands in mental ability compared to his/her peer group. IQ refers to mental age.*

☐ Normal Intellectual Ability (IQ > 80) Borderline Intellectual Ability (IQ 70-80)

☐ Mild Intellectual Disability (IQ 50-69) Moderate Intellectual Disability (IQ 35-49)

☐ Severe Intellectual Disability (IQ 21-34) Profound Intellectual Disability (IQ <20)

☐ Not known

**Developmental Quotient (DQ) score (patient below 6 years of age):**
**DQ is a numerical indicator of a child’s growth to maturity across a range of psychosocial competencies. It is an overall developmental score that relates to performance in four domains: motor skills, language use, adaptative behaviour and personal-social.*

☐ Normal Intellectual Ability

☐ Mild-Moderate Intellectual Disability

☐ Severe-Profound Intellectual Disability

☐ Not known

Has the patient had a formal evaluation of intelligence by a professional using neuropsychological tests in the last six months?	YES ☐	NO ☐	NOT SURE ☐

**In the last three months, has the patient had any of the following?**

a. Repeating words or phrases over and over again

YES ☐	NO ☐	NOT SURE ☐

b. Poor eye contact	YES ☐	NO ☐	NOT SURE ☐
c. Difficulties getting on with other people of similar age	YES ☐	NO ☐	NOT SURE ☐
d. Repetitive behaviours, such as doing the same thing over and over again	YES ☐	NO ☐	NOT SURE ☐
e. Very rigid or inflexible about how to do things or not liking change in routines

YES ☐	NO ☐	NOT SURE ☐

f. Overactivity/hyperactivity, such as being constantly on the go	YES ☐	NO ☐	NOT SURE ☐
g. Attention deficit, such as not concentrating well, getting distracted	YES ☐	NO ☐	NOT SURE ☐
h. Restlessness or fidgetiness, such as wriggling, squirming or balance continuously	YES ☐	NO ☐	NOT SURE ☐
i. Impulsivity, such as butting in, not waiting turn	YES ☐	NO ☐	NOT SURE ☐
j. Memory problems, such as not remembering things that have happened	YES ☐	NO ☐	NOT SURE ☐
k. Dual-tasking/multi-tasking, such as doing two tasks at the same time or not paying attention while doing another task

YES ☐	NO ☐	NOT SURE ☐

l. Problems with visuo-spatial tasks, such as solving puzzles or using building blocks	YES ☐	NO ☐	NOT SURE ☐
m. Deficits in executive skills, such as difficulties with planning, organizing, flexible thinking	YES ☐	NO ☐	NOT SURE ☐
n. Getting disoriented in time, such as not knowing the time of the day

YES ☐	NO ☐	NOT SURE ☐

o. Getting disoriented in space, such as not knowing the location

YES ☐	NO ☐	NOT SURE ☐

p. Stereotypies, such as hand flapping, body rocking, head banging, spinning in circles, hand biting, hand rubbing etc…

YES ☐	NO ☐	NOT SURE ☐

***If you answered YES to any of the above:***

Please, mark all the symptoms for which the patient has received a formal diagnosis or assessment by a professional (*multiple choice*):

☐ Repeating words or phrases

☐ Poor eye contact

☐ Difficulties getting on with other people of similar age

☐ Repetitive behaviours

☐ Very rigid or inflexible about how to do things or changes in routines

☐ Overactivity/hyperactivity

☐ Attention deficit

☐ Restlessness or fidgetiness

☐ Impulsivity

☐ Memory problems

☐ Dual-tasking/multi-tasking

☐ Problems with visuo-spatial tasks

☐ Deficits in executive skills

☐ Getting disoriented in time

☐ Getting disoriented in space

☐ Stereotypies

Please, specify which of the above symptoms the patient has had professional support for in the last six months (*multiple choice*):

☐ Repeating words or phrases

☐ Poor eye contact

☐ Difficulties getting on with other people of similar age

☐ Repetitive behaviours

☐ Very rigid or inflexible about how to do things or changes in routines

☐ Overactivity/hyperactivity

☐ Attention deficit

☐ Restlessness or fidgetiness

☐ Impulsivity

☐ Memory problems

☐ Dual-tasking/multi-tasking

☐ Problems with visuo-spatial tasks

☐ Deficits in executive skills

☐ Getting disoriented in time

☐ Getting disoriented in space

☐ Stereotypies

**In the last three months, has the patient had any of the following?**

a. Anxiety (including panic, phobia, separation anxiety disorder)

YES ☐	NO ☐	NOT SURE ☐

b. Depressed mood or depressive disorder (including apathy)

YES ☐	NO ☐	NOT SURE ☐

c. Extreme shyness or social withdrawal

YES ☐	NO ☐	NOT SURE ☐

d. Mood swings

YES ☐	NO ☐	NOT SURE ☐

e. Aggressive outbursts or temper tantrums

YES ☐	NO ☐	NOT SURE ☐

f. Self-injury, such as self-hitting, self-biting, self-scratching

YES ☐	NO ☐	NOT SURE ☐

g. Psychotic symptoms, including delusions, hallucinations, schizophrenia, bipolar disorder

YES ☐	NO ☐	NOT SURE ☐

h. Autism Spectrum Disorder (ASD), including autism, Asperger’s, etc.

YES ☐	NO ☐	NOT SURE ☐

i. Attention Deficit Hyperactivity Disorder (ADHD)

YES ☐	NO ☐	NOT SURE ☐

j. Obsessive Compulsive Disorder (OCD)

YES ☐	NO ☐	NOT SURE ☐

***If you answered YES to any of the above:***

Please, specify for which of the above there is a formal diagnosis or assessment by a professional (*multiple choice*):

☐ Anxiety (including panic, phobia, separation anxiety disorder)

☐ Depressed mood or depressive disorder (including apathy)

☐ Extreme shyness or social withdrawal

☐ Mood swings

☐ Aggressive outbursts or temper tantrums

☐ Self-injury, such as self-hitting, self-biting, self-scratching

☐ Psychotic symptoms, including delusions, hallucinations, schizophrenia, bipolar disorder

☐ Autism Spectrum Disorder (ASD), including autism, Asperger’s, etc.

☐ Attention Deficit Hyperactivity Disorder (ADHD)

☐ Obsessive Compulsive Disorder (OCD)

Please, specify for which of the above the patient has had professional support in the last six months (*multiple choice*):

☐ Anxiety (including panic, phobia, separation anxiety disorder)

☐ Depressed mood or depressive disorder (including apathy)

☐ Extreme shyness or social withdrawal

☐ Mood swings

☐ Aggressive outbursts or temper tantrums

☐ Self-injury, such as self-hitting, self-biting, self-scratching

☐ Psychotic symptoms, including delusions, hallucinations, schizophrenia, bipolar disorder

☐ Autism Spectrum Disorder (ASD), including autism, Asperger’s, etc.

☐ Attention Deficit Hyperactivity Disorder (ADHD)

☐ Obsessive Compulsive Disorder (OCD)

### MOTOR SKILLS

**Mobility**:

☐ needs a wheelchair

☐ needs significant support

☐ has some mobility difficulty

☐ is completely mobile

**In the last three months, has the patient had any of the following?**

a. Problems in balance	YES ☐	NO ☐	NOT SURE ☐
b. Problems in coordination (coordination consists of orchestrated movements of multiple body parts required to accomplish complex actions, like walking or grab an object from a closet)

YES ☐	NO ☐	NOT SURE ☐

c. Excessive flexion of the hips, knees, or ankles (Crouch gait)	YES ☐	NO ☐	NOT SURE ☐
d. Inability to coordinate voluntary muscular movements causing problems in walking on a straight line (Ataxia)	YES ☐	NO ☐	NOT SURE ☐
e. Tremor [shaking of the hands], bradykinesia [slowed movements], rigidity and/or postural instability (Parkinsonism)	YES ☐	NO ☐	NOT SURE ☐

***If you answered YES to any of the above:***

Has the patient received professional support for it (e.g., physical therapy) in the last six months?

YES ☐	NO ☐	NOT SURE ☐

Please specify for which of the above the patient has received professional support in the last six months (*multiple choice*):

☐ Problems in balance

☐ Problems in coordination

☐ Excessive flexion of the hips, knees, or ankles (Crouch gait)

☐ Inability to coordinate voluntary muscular movements causing problems in walking on a straight line (Ataxia)

☐ Tremor [shaking of the hands], bradykinesia [slowed movements], rigidity and/or postural instability (Parkinsonism)

### DAILY LIFE

**Let’s talk now about daily life.**

**Self-care in daily activities**

☐ The patient is dependent on others

☐ The patient has some self-care skills

☐ The patient is independent

**Eating**

☐ The patient is dependent on others

☐ The patient has some self-care skills

☐ The patient is independent

**Quantity of eating**

☐ Too little

☐ Normal

☐ Too much

**Type of food** *(multiple choice)*

☐ Very selective

☐ No issues

☐ Eating unusual things

**Swallowing problems**

☐ Yes

☐ No

☐ Not sure

**Sleeping:**

a. Problems falling asleep

YES ☐	NO ☐	NOT SURE ☐

b. Problems waking up

YES ☐	NO ☐	NOT SURE ☐

c. Waking up not related to seizures

YES ☐	NO ☐	NOT SURE ☐

d. Waking up due to seizures

YES ☐	NO ☐	NOT SURE ☐

e. Early waking up

YES ☐	NO ☐	NOT SURE ☐

f. Sleep apnoea (breathing stops repeatedly during sleep for more than 15 seconds)

YES ☐	NO ☐	NOT SURE ☐

g. Taking sleep medications (e.g., melatonin)

YES ☐	NO ☐	NOT SURE ☐

h. The patient is sleeping in the same room of the parents, but in his/her own bed

YES ☐	NO ☐	NOT SURE ☐

i. The patient is sleeping in the bed of the parents

YES ☐	NO ☐	NOT SURE ☐

j. The patient is sleeping under the supervision of an alarming system (e.g., camera)

YES ☐	NO ☐	NOT SURE ☐

***If you answered YES to any of the above:***

Please, specify for which of the above there is a formal diagnosis or assessment by a professional (*multiple choice*):

☐ Problems falling asleep

☐ Problems waking up

☐ Waking up without seizures

☐ Waking up because of seizures

☐ Early waking up

☐ Sleep apnoea (breathing stops repeatedly during sleep for more than 15 seconds)

☐ Taking medications for sleeping (e.g., melatonin)

☐ The patient is sleeping in the same room of the parents, but in his/her own bed

☐ The patient is sleeping in the bed of the parents

☐ The patient is sleeping under the supervision of an alarming system (e.g., camera)

Please, specify for which of the above the patient has had professional support in the last six months (*multiple choice*):

☐ Problems falling asleep

☐ Problems waking up

☐ Waking up without seizures

☐ Waking up because of seizures

☐ Early waking up

☐ Sleep apnoea (breathing stops repeatedly during sleep for more than 15 seconds)

☐ Taking medications for sleeping (e.g., melatonin)

☐ The patient is sleeping in the same room of the parents, but in his/her own bed

☐ The patient is sleeping in the bed of the parents

☐ The patient is sleeping under the supervision of an alarming system (e.g., camera)

Please, specify if a polysomnography (sleep study) has been performed:

YES ☐	NO ☐	NOT SURE ☐

### QUALITY OF LIFE (QoL) OF THE FAMILY

**Let’s talk now about the quality of life of caregivers, including both parents and siblings.**

**Has the QoL of the family somehow changed?**

a. Life of siblings has changed completely

YES ☐	NO ☐ NO SIBLINGS ☐

b. ☐☐Very high levels of stress between parents leading to significant	relationship difficulties

YES ☐	NO ☐

c. Very high levels of stress within the family, for instance between siblings	YES ☐	NO ☐
d. Family life has been significantly altered because of Dravet syndrome

YES ☐	NO ☐

**If YES, in which area?**

☐ School (siblings)

☐ Work (parents)

☐ Leisure time

☐ Leisure time (parents)

☐ Holidays

☐ Relationships with relatives

☐ Sleep (parents)

☐ Sleep (siblings)

☐ Social time (siblings)

☐ Social time (parents)

How much have these bothered, troubled, or distressed you/your family in comparison to before Dravet syndrome onset?

*(1, not at all; 2, slightly; 3, somewhat; 4, moderately; 5, extremely)*

1	2	3	4	5

Have any family members received psychological support due to Dravet syndrome?

YES ☐	NO ☐	NOT SURE ☐

If YES, who receive psychological support?

Father ☐	Mother ☐	Sibling(s) ☐

### FOR ALL THE ITEMS LISTED BELOW, PLEASE RATE YOUR LEVEL OF CONCERN

(0, not important at all; 10, super important)

Cognition (patient)

0 1 2 3 4 5 6 7 8 9 10

Behaviour (patient)

0 1 2 3 4 5 6 7 8 9 10

Motor skills (patient)

0 1 2 3 4 5 6 7 8 9 10

Daily life (patient)

0 1 2 3 4 5 6 7 8 9 10

Eating (patient)

0 1 2 3 4 5 6 7 8 9 10

Sleeping (patient)

0 1 2 3 4 5 6 7 8 9 10

Quality of Life of the family

0 1 2 3 4 5 6 7 8 9 10

### DO YOU HAVE ANY OTHER WORRIES FOR THE PATIENT THAT WE HAVE NOT TALKED ABOUT AS WE WENT THROUGH THE CHECKLIST?

YES ☐	NO ☐

If YES, please ADD here:

### WOULD YOU LIKE TO SEND A REPORT TO YOUR PHYSICIAN?

Enter your physician’s email address in case you would like him/her to send him/her a report with the results from this questionnaire:

The DANCE Checklist is distributed under the terms of the Creative Commons Attribution-NonCommercial-NoDerivatives 4.0 International Licence (CC BY-NC-ND 4.0, http://creativecommons.org/).

The DANCE Checklist may be copied, reproduced and used in paper format for non-commercial purposes provided that the original work is properly credited and that no changes are made to the checklist. For any enquiries regarding use of the DANCE Checklist, please contact Dravet Syndrome Foundation Spain
